# Integrative multi-stage deep learning framework for ovarian tumor ultrasound classification with explainability and confidence estimation

**DOI:** 10.3389/fmed.2025.1760167

**Published:** 2026-02-24

**Authors:** Shtwai Alsubai, Ahmad Almadhor, Abdullah Al Hejaili

**Affiliations:** 1 College of Computer Engineering and Sciences, Prince Sattam bin Abdulaziz University, Al-Kharj, Saudi Arabia; 2 Department of Computer Engineering and Networks, College of Computer and Information Sciences, Jouf University, Sakaka, Saudi Arabia; 3 Computer Science Department, Faculty of Computers & Information Technology, University of Tabuk, Tabuk, Saudi Arabia

**Keywords:** deep learning, EfficientNet, explainable AI, MMOTU dataset, ovarian cancer, tumor classification, ultrasound imaging

## Abstract

**Introduction:**

Ovarian cancer is a major diagnostic problem because it is asymptomatic in its early stages and requires subjective interpretation of ultrasound images.

**Methods:**

This research presents the EfficientOvaNet framework, a deep learning-based model for classifying ovarian tumors using ultrasound images, trained on the Multi-Modality Ovarian Tumor Ultrasound (MMOTU) dataset. The framework employs a two-branch EfficientNet-B3 architecture that combines Region-of-Interest (ROI) features with global contextual information. Sophisticated preprocessing, data augmentation, and class-imbalance control using weighted Focal Loss are applied. Five-fold cross-validation is used for performance evaluation. Explainable methods, including Grad-CAM, Monte Carlo Dropout uncertainty estimation, and t-distributed Stochastic Neighbor Embedding (t-SNE)-based feature visualization, are incorporated to ensure interpretability.

**Results:**

The five-fold cross-validation yields a mean accuracy of 91.9%, an F1-score of 91.9%, and an AUC of 0.98, indicating better performance than baseline models.

**Discussion:**

EfficientOvaNet increases diagnostic accuracy and reduces subjectivity in ultrasound-based ovarian tumor classification. By improving interpretability and credibility, the framework has the potential to support timely intervention and individualized treatment, which may improve survival rates in the management of ovarian cancer.

## 1 Introduction

Ovarian cancer is among the deadliest gynecologic cancers in the world, being listed as the eighth most frequent cause of cancer-related death among females (1). In 2020, GLOBOCAN reported over 314,000 new cases and 207,000 deaths globally (2). Nevertheless, despite the advances in the care of ovarian cancer, the overall 5-year survival rate of ovarian cancer is only about 47% compared to breast cancer (90%) under the same conditions (3). This is mainly due to late diagnosis when the disease is in an early stage, with symptoms that are either minimal or nonspecific, such as abdominal pain, bloating, and urinary frequency (4). As a result, almost 70% of cases are diagnosed at a late stage, when they have become metastatic.

Ovarian cancer has a multifactorial etiology that is a complex interaction among genetic, hormonal, and environmental factors. Risk factors include mutations in the *BRCA1* and *BRCA2* genes, family history of ovarian/breast cancer, nulliparity, late pregnancy, post-menopausal hormone therapy, endometriosis, and obesity (5). The disease is exquisitely heterogeneous and includes subtypes high-grade serous carcinoma, low-grade serous carcinoma, endometrioid carcinoma, and clear cell carcinoma, with varying genetic composition, histopathology, and response to therapy for each variant (6).

Ovarian cancer is a major clinical challenge that has not been thoroughly addressed because of the constraints of the traditional screening procedures. The existing methods, such as pelvic examinations, transvaginal ultrasound (US), and serum biomarkers, including cancer antigen 125 (CA-125), are not sufficiently sensitive or specific to detect the disease at an early stage (7). Despite the emergence of sophisticated imaging techniques, including computed tomography (CT) and magnetic resonance imaging (MRI), which can provide detailed structural data, these techniques are often costly and time-consuming. They cannot be performed during routine screening. Although advanced imaging modalities such as computed tomography (CT) and magnetic resonance imaging (MRI) can provide more detailed structural data, they are costly and time-consuming. They cannot be used routinely for screening (8). The diagnostic gold standard is histopathological examination, an invasive method that is prone to interobserver variability.

In recent years, artificial intelligence (AI) has emerged as a transformative medical diagnostic tool, enabling objective, automated, and repeatable evaluation of imaging data, at the expense of procedural automation and computerization (9, 10). In the field of AI, deep learning, especially convolutional neural networks (CNNs), has shown impressive results in processing intricate medical images to surpass the performance of traditional feature-engineered models in different tasks such as classification, detection, and segmentation (11). Deep learning models have been used in ovarian cancer imaging to detect tumors, classify lesions, and predict malignancy across ultrasound, MRI, and CT images (12).

Specifically, ultrasound is a non-invasive, real-time, cost-effective diagnostic modality for evaluating ovarian tumors. Nonetheless, manual interpretation of ultrasound images is highly operator-dependent and inherently variable (13). Deep learning frameworks, when combined with ultrasound imaging, can improve diagnostic accuracy by automatically detecting tumor regions, differentiating between benign and malignant tumors, and reducing subjectivity in clinical decision-making (10).

The objective of this study is to develop an deep learning framework (i.e., EfficientOvaNet) for automated classification of ovarian tumors from ultrasound images. The proposed framework leverages transfer learning, advanced preprocessing, and explainable AI techniques to achieve accurate and interpretable diagnostic predictions. By focusing on model efficiency, interpretability, and clinical applicability, this research aims to contribute to the early and reliable detection of ovarian malignancies, thereby supporting clinicians in timely diagnosis and personalized treatment planning.

The major contributions of this study are summarized as follows:

- Development of EfficientOvaNet framework, a novel dual-branch EfficientNet-B3 model that synergistically processes ROI-cropped tumor regions and full ultrasound images to capture fine-grained local features and global anatomical context, achieving state-of-the-art classification accuracy on the MMOTU dataset.
- The study implements a robust preprocessing and augmentation pipeline that leverages ROI extraction, intensity normalization, and advanced methods such as MixUp and CutMix, alongside weighted Focal Loss to address class imbalance and improve generalization in noisy ultrasound data. It ensures reliable and interpretable predictions through five-fold cross-validation (accuracy, F1-score, AUC) and explainability tools, including Grad-CAM, MC-Dropout uncertainty, and t-SNE embeddings, for clinician-trustworthy deployment.
- Finally, the research demonstrates clinical relevance by showing high discriminative power (AUC = 0.98) and low calibration error, positioning the framework as a reliable aid for early ovarian tumor detection, reducing diagnostic variability, and supporting personalized oncology care.

The rest of the paper is structured as follows. Section 2 discusses the related work on Ovarian cancer detection. Followed by the proposed framework in Section 3. Section 4 presents the experiential analysis and results. Finally, Section 5 concludes the paper.

## 2 Related work

This section discusses the related work on Ovarian cancer detection. Garg (14) compared six deep learning architectures with distinct backbones to evaluate their performance and radiomic stability on 1,469 two-dimensional (2D) ultrasound images from the Multi-Modality Ovarian Tumor Ultrasound (MMOTU) dataset. The models being assessed included U-Net++, DeepLabV3+, U-Net, SegFormer, FPN, and LinkNet. The performance was evaluated using Dice and IoU scores, precision, recall, specificity, size error, and the 95th percentile Hausdorff distance (HD95). Radiomic stability was assessed by extracting 102 quantitative features and calculating Pearson correlation coefficients between predicted and ground-truth masks. The results showed that all models performed well, achieving average Dice scores of 0.9311, 0.9259, 0.9277, 0.9271, 0.9280, and 0.9213 for U-Net++, DeepLabV3+, U-Net, SegFormer, FPN, and LinkNet, respectively. Radiomic correlations were also high, ranging from 0.9400 to 0.9518, and were statistically significant across models (*p* < 0.05). U-Net++ achieved the best Dice, IoU, precision, HD95, and size error, while SegFormer demonstrated the highest radiomic stability.

Pham et al. (15) proposed a comparative study on ovarian tumor segmentation of ultrasound images using the state-of-the-art models (PSPNet, U-Net, DANet, Deeplabv3, and PSANet) and tested them on the MMOTU dataset. The authors created pixel-level annotations for eight tumor types, unlike earlier MMOTU-based literature, which used binary segmentation to estimate tumor size, shape, and disease type concurrently. The experimental results showed that DANet achieved the highest average accuracy of 71.65%. In contrast, PSANet segmented the chocolate cysts category with an average IoU of 96.33%, demonstrating excellent model-specific performance across tumor subtypes. Siahpoosh et al. (16) proposed a U-Net-based network, PCU-Net, for ovarian tumor segmentation that leverages ConvMixer and Pyramid Dilated Convolution (PDC) modules. The ConvMixer component employed a global context with a large-kernel convolution to capture global information. In contrast, the PDC module refined multi-scale feature extraction via parallel dilated convolution with different dilation rates. The model was also designed to use fewer parameters than the original U-Net and to enhance contextual learning. MMOTU data were tested using the proposed method, in which PCU-Net outperformed U-Net by 4.23% in Intersection over Union (IoU) and 2.99% in Dice Similarity Coefficient (DSC), indicating superior segmentation performance.

Deepika and Chitra (17) developed a deep learning-based U-Net model for automated ovarian tumor segmentation from ultrasound images to enhance the accuracy of diagnoses and clinical decisions. The model was trained and tested on the MMOTU dataset using a Binary Cross-Entropy with Intersection over Union (BCE-IoU) loss function to improve boundary accuracy. The model achieved an IoU of 0.7592, a Dice coefficient of 0.8234, and a pixel-wise accuracy of 91.5%, outperforming those of comparative models, including ResNet-based segmentation, DeepLabV3+, and Attention U-Net. These results validated the U-Net's ability to detect tumor regions in low-contrast, noisy ultrasound images. The authors proposed a way to further improve it by using attention mechanisms, multimodal data fusion, and real-time clinical deployment. Chen et al. (18) constructed a 2D ovarian-tumor ultrasound image inpainting dataset by finely annotating symbols and artifacts in the MMOTU dataset, and proposed a novel Mask-Guided Generative Adversarial Network (MGGAN) to remove such symbols. The generator integrated an attention mechanism to prioritize valid lesion information while ignoring unwanted symbols, aided by Fast Fourier Convolution (FFC) and residual blocks to expand the global receptive field. The model achieved high-quality pixel-level inpainting at both 256 × 256 and 512 × 512 resolutions. At 256 × 256, MGGAN achieved SSIM = 0.9246, FID = 22.66, and LPIPS = 0.07806, while at 512 × 512, it reached SSIM = 0.9208, FID = 25.52, and LPIPS = 0.08300. Moreover, segmentation performance improved notably on the cleaned images. U-Net's accuracy increased from 71.51 to 76.06%, and PSPNet's from 61.13 to 66.65%, confirming the efficacy of symbol removal in improving ovarian tumor diagnosis and segmentation accuracy.

Rasheed et al. (19) study proposed an advanced framework that integrates Convolutional Neural Networks (CNNs) with a hybrid attention mechanism for the classification of primary brain tumors, including glioma, meningioma, pituitary tumors, and non-tumor cases. The proposed approach was extensively evaluated using benchmark datasets reported in the literature and compared with several well-established pre-trained architectures, including Xception, ResNet50V2, DenseNet201, ResNet101V2, and DenseNet169. Experimental results demonstrated outstanding performance, achieving an accuracy of 98.33%, with precision and recall values of 98.30% and an F1-score of 98.20%. Ahmad et al. (20) introduced a patient-specific seizure prediction framework integrated with the Consumer Internet of Things (CIoT) for innovative healthcare applications. The approach extracts and fuses handcrafted and deep features, which are then modeled using a Bidirectional Long Short-Term Memory (BiLSTM) network to capture the temporal characteristics of EEG signals. An attention mechanism is employed to reduce feature dimensionality, while the CIoT module enables real-time seizure prediction and alert transmission through a cloud-based platform. Evaluation using Leave-One-Out cross-validation demonstrated consistent performance across multiple seizure types, achieving an accuracy of 91.39%, sensitivity of 91.30%, specificity of 90.06%, and a false positive rate of 0.12 for a seizure alarm horizon of 10 min.

Ahmad et al. (21) proposed an interpretable multi-view feature learning framework (IMV-FL) for EEG-based seizure detection. The approach transforms time-domain EEG signals into frequency-domain representations using the Discrete Fourier Transform (DFT) and extracts spatial and temporal features via ResNet and LSTM models. Feature compression is performed using a Deep Neural Network (DNN), followed by multi-view feature fusion and selection through a Mutual Information-Based Feature (MIBF) algorithm. Classification is achieved with a Stacking Ensemble Classifier (SAEC), while SHapley Additive exPlanations (SHAP) enhance clinical interpretability. Evaluation on the CHB-MIT Scalp and Bonn EEG datasets demonstrated that IMV-FL outperforms single-view and state-of-the-art methods by 2%–3% across accuracy, sensitivity, specificity, and F1-score, highlighting its effectiveness for clinical seizure detection.

## 3 Proposed framework

**Figure 1:**
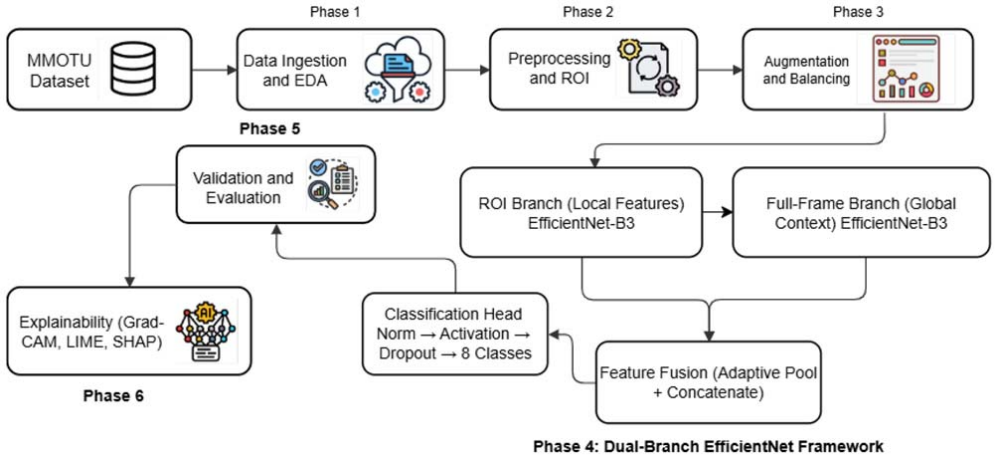
Overview of the proposed EfficientOvaNet framework for ovarian tumor ultrasound classification.

This section elaborates on the overall workflow of the proposed deep learning framework, termed EfficientOvaNet, developed for automated ultrasound-based classification of ovarian tumors. The framework operates through six sequential phases, encompassing data ingestion, preprocessing, augmentation, model design, validation, and explainability. Figure 1 illustrates the architectural pipeline, highlighting each processing stage and its data flow, while Algorithm 1 presents the stepwise pseudocode outlining the operational logic of the proposed EfficientOvaNet model.

Algorithm 1 Proposed EfficientOvaNet framework for ultrasound tumor classification. 
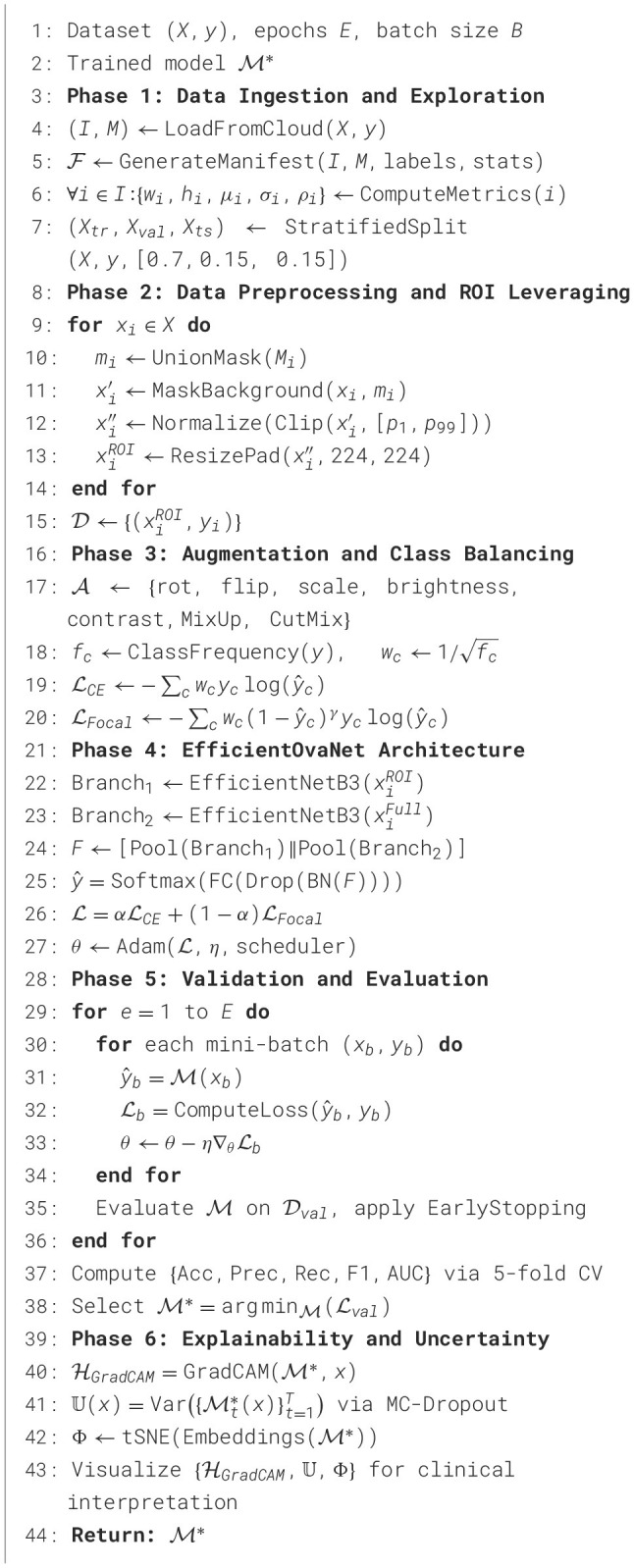

### 3.1 Experimental dataset

This study uses the publicly available Multi-Modality Ovarian Tumor Ultrasound (MMOTU) dataset (22), created by Zhao et al. at Beijing Shijitan Hospital, Capital Medical University. The MMOTU dataset has been developed to support research focused on ovarian tumor segmentation and classification across various imaging settings. It consists of ultrasound images of several devices, in both *B-mode conventional ultrasound* (*OTU_2D*) and *contrast-enhanced ultrasound* (*OTU_CEUS*) modes, spanning a wide range of texture variations and intensity distributions. The dataset contains 1,469 two-dimensional (2D) grayscale ultrasound scans in the OTU_2D subset and 170 images in the CEUS subset, each with expert radiologist annotations. The images are also accompanied by a pixel-wise binary mask outlining the tumor region and a global class label indicating whether the tumor is benign or malignant. The dataset is suitable for supervised learning, semantic segmentation, and unsupervised cross-domain adaptation tasks, given these annotations. The MMOTU dataset is rich in metadata, including the acquisition modality, image resolution, and scanner source, which facilitates model generalization and domain transfer. Each image was de-identified and is available for research use via the official GitHub repository (23). The variety of imaging modalities and diagnostic presentations in the dataset makes it a standard resource for analyzing ovarian tumors in medical imaging and computer vision. The summary of this dataset is provided in Table 1.

**Table 1:** Summary of MMOTU dataset characteristics.

**Attribute**	**OTU_2D subset**	**OTU_CEUS subset**	**Total**
Number of images	1,469	170	1,639
Image modality	Conventional ultrasound	Contrast-enhanced ultrasound	–
Annotation type	Binary mask (tumor ROI)	Binary mask (tumor ROI)	–
Tumor categories	Benign/malignant	Benign/malignant	–
Image dimension range	400 × 800 px	400 × 800 px	–
Acquisition site	Beijing Shijitan Hospital	Beijing Shijitan Hospital	–
Annotation source	Expert radiologists	Expert radiologists	–
Public availability	https://github.com/cv516Buaa/MMOTU_DS2Net		

### 3.2 Phase 1: data ingestion and exploration

The Data Ingestion phase serves as the foundational stage of the proposed framework, responsible for acquiring raw ultrasound data, organizing heterogeneous inputs, validating annotations, and generating structured metadata required for reliable deep learning training. This phase ensures that all subsequent processing steps operate on a consistent, traceable, and quality-verified dataset.

The first phase of the proposed framework involved ingesting and systematically exploring the ultrasound tumor dataset to organize it, make it available, and prepare it for deep learning processing. The dataset was programmatically retrieved from cloud storage and mounted into the local processing environment, where directory paths were explicitly defined for raw ultrasound images, segmentation masks (annotations), and output logs. All intermediate and processed data were stored in organized directories to enable traceability throughout subsequent phases. An automated directory traversal and validation procedure was employed to identify and index all available data files. The system recursively searched predefined image and annotation folders, with a fallback scan of the root directory to ensure no files were omitted. This process retrieved 1,469 image files and 4,407 mask files. File path previews were generated to verify directory consistency and dataset completeness.

Label information was extracted from text files that mapped image identifiers to class labels, and the data were converted into structured metadata, enabling straightforward linking of each image to its class label. To ensure correct correspondence between ultrasound images and their segmentation masks, a robust lookup table was constructed using filename stem matching, incorporating exact, prefix-based, and containment-based matching strategies. This procedure ensured that all valid masks were correctly associated with their corresponding images. For each image, descriptive metadata including spatial resolution (width and height), number of associated masks, and pixel intensity statistics (mean and standard deviation) were computed. These attributes were consolidated into a centralized manifest file that functioned as a comprehensive dataset index and quality-control reference.

**Figure 2:**
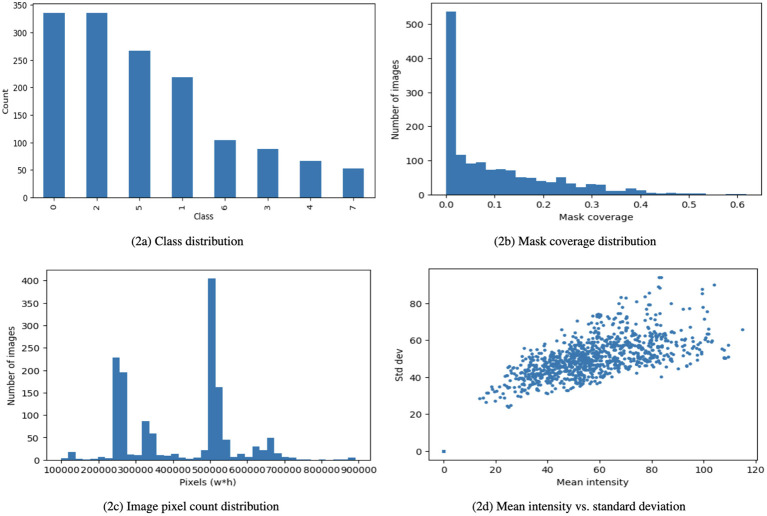
Phase-1 exploratory data analysis of the MMOTU dataset: **(a)** class distribution, **(b)** mask coverage distribution, **(c)** image pixel count distribution, and **(d)** mean intensity vs. standard deviation.

Exploratory analysis measured the mask coverage ratio, defined as the proportion of tumor pixels relative to the total image area, to identify samples that were under- or over-annotated. Image quality assessments based on pixel intensity histograms were used to detect low-contrast, overexposed, or noisy images. Additionally, patient-level identifiers embedded in filenames were analyzed to assess inter-sample dependencies. As no repeated patient instances were detected, a stratified random-splitting strategy was adopted rather than patient-wise grouping. The dataset was partitioned into training, validation, and test subsets using a 70:15:15 ratio, with stratified sampling to preserve class distributions across splits. Each subset retained its corresponding metadata records to maintain full traceability between raw inputs, annotations, and experimental outcomes. Exploratory Data Analysis (EDA) visualized class distribution Figure 2a, mask coverage Figure 2b, image size variability Figure 2c, and pixel intensity characteristics Figure 2d. Finally, qualitative validation was performed by superimposing segmentation masks onto their corresponding ultrasound images to confirm spatial alignment and annotation accuracy visually. This phase ensured the dataset is clean, consistent, and reliable for model training, providing crucial insights into class balance, mask representation, and image characteristics.

Figure 2a shows the distribution of classes and the imbalance between the classes. Specifically, classes 0 and 2 have the largest sample sizes, followed by classes 5 and 1, which have moderate representation, whereas classes 6, 3, 4, and 7 have relatively few samples. This uneven distribution indicates a class-imbalanced dataset, which could bias the model toward the majority classes during training and negatively affect its performance on underrepresented classes. In contrast, Figure 2b depicts the mask coverage distribution, which shows the proportion of each image covered by a mask. The histogram shows that most images have low mask coverage (below 0.1), while only a few exhibit higher coverage. This right-skewed distribution suggests that the regions of interest occupy a small portion of most images. Such sparsity may make it challenging for the model to effectively learn from limited labeled areas, underscoring the need for strategies such as data augmentation, loss weighting, or focused sampling to improve training balance and segmentation accuracy. Figure 2c shows the distribution of image pixel counts, indicating that the dataset contains images with varying resolutions. The multiple peaks in the histogram indicate distinct image-size groups, suggesting that the data were collected from different sources or imaging settings. This variability in image dimensions implies that resizing or normalization is necessary before model training to ensure consistent input sizes and stable learning behavior. Figure 2d depicts the relationship between image mean intensity and standard deviation, showing a positive correlation. Images with higher mean intensity generally exhibit greater pixel-value variation, indicating greater contrast or richer texture. In comparison, darker images (with lower mean intensity) tend to have lower standard deviation. This relationship provides insight into the overall brightness and contrast diversity in the dataset, which can influence model generalization and robustness.

### 3.3 Phase 2: data preprocessing and ROI leveraging

The second phase focused on refining and standardizing the dataset produced in Phase 1 to ensure uniformity and quality before model training. The manifest file, which contains paths to images, masks, and class labels for all 1,469 samples, was used for structured preprocessing. The workflow emphasized region-of-interest (ROI) extraction, intensity normalization, and size standardization to enhance focus on tumor regions while minimizing background variability. Among three ROI modes, Mode A (crop + pad + resize), Mode B (mask background), and Mode C (dual-stream saving), Mode B was selected for this study, wherein non-tumor regions were masked out to highlight the lesion area. Each image was resized with aspect-ratio preservation and center-cropped to 224 × 224 pixels. Intensity values were clipped to the 1st-99th percentile range to enhance contrast and normalized to a fixed scale. Multiple binary masks corresponding to a single image were merged into a unified mask, ensuring complete lesion representation. Subsequently, the image was cropped around the ROI bounding box and padded to a square shape if necessary. Processed images were saved in the Phase 2 output directory using standardized naming conventions. Metadata comprising image identifiers, original and processed paths, class labels, and ROI mode were logged systematically. For Mode C, an additional full-image version was retained for dual-branch training. This phase produced a clean, intensity-normalized, and ROI-focused dataset optimized for deep learning applications.

### 3.4 Phase 3: data augmentation and class imbalance handling

Phase 3 expanded the dataset's diversity and addressed inherent class imbalance while preparing training-ready data pipelines. Configuration parameters such as image size, batch size, and computation device were specified, and the processed manifest and split files were loaded to construct consistent train, validation, and test sets. A multi-stage augmentation pipeline was implemented, combining geometric transformations (rotation, scaling, flipping) with photometric adjustments (brightness, contrast, gamma correction). Advanced strategies such as MixUp and CutMix were incorporated to further improve generalization and model regularization. Augmentations were applied probabilistically to training samples, while validation and test data underwent only deterministic resizing and normalization to maintain evaluation integrity. To mitigate class imbalance, class frequencies were analyzed, and corresponding weights were computed as the inverse square root of class occurrence. Weighted sampling ensured proportional representation of minority classes within each mini-batch. Additionally, weighted Cross-Entropy and Focal Loss functions were defined to reduce bias toward dominant classes. Focal Loss dynamically downweights easy samples, thereby improving the sensitivity to the minority class. Augmented image samples were visually verified for realism and correctness. A preliminary evaluation using a lightweight pretrained backbone confirmed proper data formatting, augmentation consistency, and label alignment. This phase established a robust, balanced, and model-ready dataset foundation through controlled augmentation and reweighting strategies.

### 3.5 Phase 4: model architecture EfficientOvaNet framework

Phase 4 introduced the proposed dual-branch deep learning framework, EfficientOvaNet, which leverages an EfficientNet-B3 backbone as its computational core. EfficientOvaNet does not introduce a novel backbone architecture; rather, it employs the EfficientNet-B3 model within a dual-branch design to integrate local and global contextual features. The Phase 2 dataset and the stratified partitions from Phase 3 were used for training (70%), validation (15%), and testing (15%). All inputs were standardized to 224 × 224 resolution with runtime augmentations applied to the training set. Class imbalance was handled by reweighting based on the inverse square root of frequencies, and Focal Loss was selected for its ability to emphasize complex and minority-class examples.

EfficientOvaNet consists of two synchronized convolutional branches: the first processing cropped ROI images to learn fine-grained tumor boundaries, and the second handling full ultrasound frames to capture global anatomical context. Both branches share an EfficientNet-B3 backbone with synchronized weights. Extracted feature maps from both streams undergo adaptive average pooling and concatenation before being passed through a fully connected classification head composed of normalization, activation, dropout, and an output layer representing eight tumor classes. Model optimization used an adaptive optimizer with cyclical learning rate scheduling to achieve stable convergence. Mixed-precision training accelerated computation, while dropout and early stopping provided additional regularization. The resulting model effectively fused local and contextual cues, achieving efficient dual-stream feature integration and high discriminative power.

## 4 Experimental analysis and results

This section presents the experimental analysis and results of the proposed EfficientOvaNet model on the MMOTU ultrasound dataset, including AUC, accuracy, F1-score, confusion matrix, ROC curve, and SHAP-based explainability analysis.

### 4.1 Phase 5: validation and evaluation

Comprehensive validation and evaluation of EfficientOvaNet were performed to assess its stability, generalization, and clinical readiness. Table 2 summarizes the five-fold cross-validation performance of the proposed model on the MMOTU dataset. A stratified five-fold cross-validation (*K* = 5) scheme was employed to ensure equitable class representation. The results demonstrate stable, consistent performance across all folds, with mean accuracies and F1-score of 0.919 and an AUC of 0.982, respectively. The low standard deviations indicate minimal variance across folds, confirming the model's robustness and reliable generalization. Moreover, the narrow 95% confidence intervals further validate the model's statistical stability, underscoring its strong discriminative capability for classifying ultrasound images of ovarian tumors.

**Table 2:** Five-fold cross-validation performance summary of the proposed EfficientOvaNet model.

**Fold**	**Accuracy (ACC)**	**F1-score**	**AUC**
1	0.901	0.900	0.970
2	0.909	0.910	0.978
3	0.935	0.938	0.985
4	0.948	0.949	0.994
5	0.900	0.901	0.983
Mean	0.919	0.919	0.982
Std. Dev.	0.0217	0.0223	0.0087
95% CI	(0.902–0.938)	(0.902–0.937)	(0.975–0.989)

**Figure 3:**
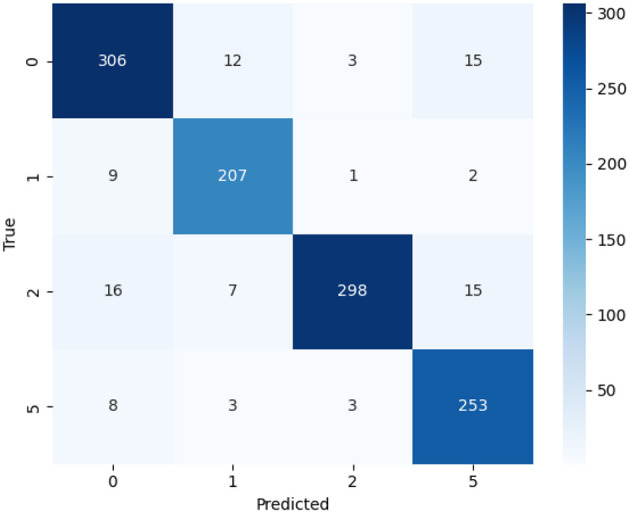
Confusion matrix of proposed EfficientOvaNet model.

Figure 3 shows the confusion matrix, which illustrates model performance for each class. Diagonal elements indicate correctly classified samples, while off-diagonal elements show misclassifications. This figure is important because it highlights specific class confusions and the overall classification strength, providing insight into areas that may require improvement. The matrix comprises four classes labeled 0, 1, 2, and 5, which denote distinct ovarian tumor categories in the MMOTU dataset. The diagonal elements indicate correctly classified instances for each class: 306 for class 0, 207 for class 1, 298 for class 2, and 253 for class 5, reflecting high classification accuracy across all categories. The off-diagonal values represent misclassified instances, highlighting areas where the model confuses one class with another (e.g., in the actual class 0 row, 12 samples were predicted as class 1, 3 as class 2, and 15 as class 5). The color intensity in the matrix visually emphasizes prediction frequency, with darker shades indicating higher counts and lighter shades indicating fewer predictions. Overall, the predominance of higher diagonal values and minimal off-diagonal errors indicates that EfficientOvaNet achieves strong discriminative performance and reliable classification accuracy across tumor classes.

**Figure 4:**
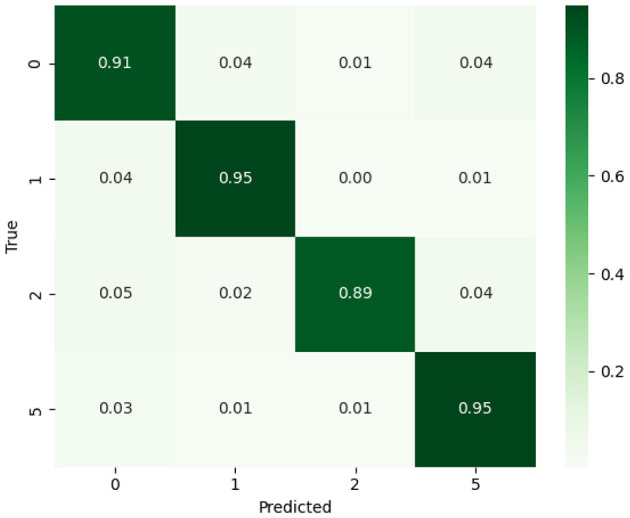
Normalized confusion matrix of the proposed EfficientOvaNet model.

Figure 4 presents the normalized confusion matrix, which communicates relative performance across classes regardless of sample size. This visualization provides an intuitive understanding of class-wise accuracy proportions, highlighting the model's robustness and consistency across imbalanced data. This graph illustrates the proportional performance of the proposed EfficientOvaNet model across tumor classes 0, 1, 2, and 5. Each cell represents the fraction of predictions relative to the total actual instances of a class, enabling a balanced comparison regardless of class size. The diagonal values indicate class-wise accuracies of 91% for class 0, 95% for class 1, 89% for class 2, and 95% for class 5, demonstrating strong overall classification performance. Off-diagonal values highlight minor misclassifications, primarily between classes 0, 2, and 5.

**Figure 5:**
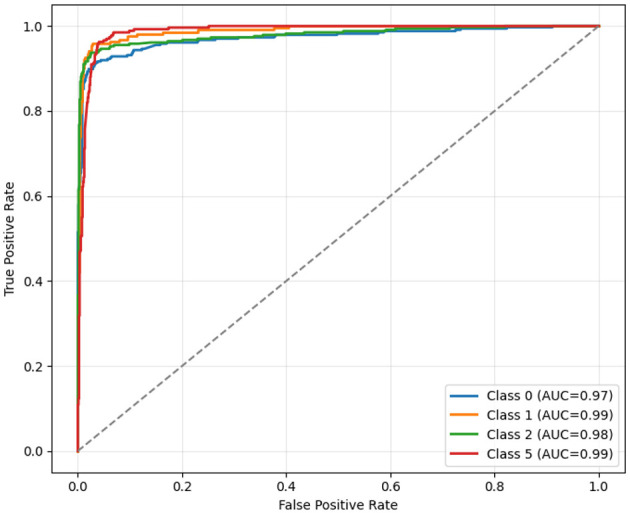
ROC curves of the proposed EfficientOvaNet model.

Figure 5 shows ROC curves, which are crucial for understanding the trade-off between sensitivity and specificity for each tumor class. The AUC values indicate the model's discriminative power, with higher curves reflecting better performance. This figure demonstrates the reliability of EfficientOvaNet in distinguishing between classes across varying decision thresholds. This evaluates the classification performance across tumor classes 0, 1, 2, and 5. The *x*-axis represents the False Positive Rate (FPR), the proportion of negative instances incorrectly classified as positive. In contrast, the *y*-axis represents the True Positive Rate (TPR), also known as Recall or Sensitivity, the proportion of actual positives correctly identified. The dashed diagonal line from (0,0) to (1,1) represents a random classifier with no predictive ability (AUC = 0.5). Each colored curve corresponds to a specific class and shows the trade-off between TPR and FPR across different threshold values. The AUC values, as shown in the legend, are 0.97 for class 0, 0.99 for class 1, 0.98 for class 2, and 0.99 for class 5, indicating excellent discriminative ability. The curves lie well above the diagonal, indicating highly accurate, favorable rates and low false-positive rates across all classes. In particular, classes 1 and 5 achieve near-perfect classification with an AUC of 0.99.

**Figure 6:**
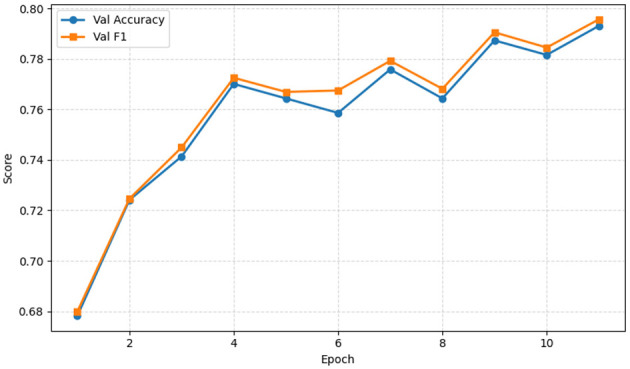
Validation accuracy and F1 over epochs of the proposed EfficientOvaNet model.

Figure 6 presents validation accuracy and F1 score over 12 training epochs, conveying the model's learning progression and consistency between overall correctness and precision-recall trade-off. This figure is important because it demonstrates effective training dynamics and generalization capability. The *x*-axis represents the number of epochs, while the *y*-axis denotes the metric values ranging approximately from 0.67 to 0.80. Validation accuracy (blue line with circles) measures the proportion of correctly predicted instances, and the validation F1 score (orange line with squares) represents the harmonic mean of precision and recall, balancing false positives and false negatives. Both metrics begin at approximately 0.68 in the 1st epoch and steadily increase as training progresses, with minor fluctuations between the 3rd and 12th epochs. By the final epoch, both metrics approach 0.80, indicating strong model performance. The close alignment between validation accuracy and F1 score demonstrates that the model maintains a consistent balance between overall correctness and the trade-off between precision and recall, reflecting effective learning and generalization during training.

**Figure 7:**
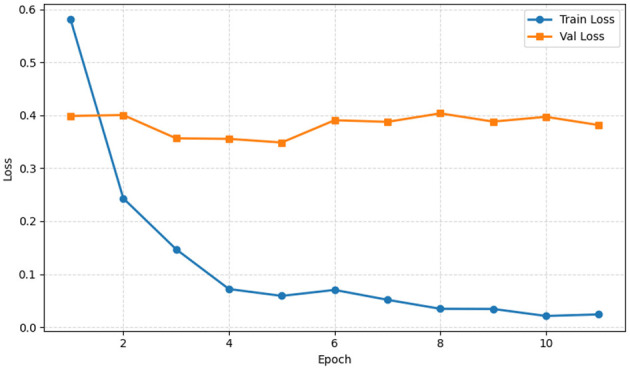
Training and validation loss of the proposed EfficientOvaNet model.

Figure 7 shows training and validation loss over 11 epochs, indicating the model's convergence behavior. The figure highlights differences between seen and unseen data, guiding readers on generalization and potential overfitting concerns. The *x*-axis represents the number of epochs, while the *y*-axis denotes the corresponding loss values ranging from 0 to 0.6. The training loss starts at approximately 0.6 and decreases rapidly during the initial epochs, gradually approaching zero by the 11th epoch, indicating effective learning and a strong fit to the training data. In contrast, the validation loss begins at approximately 0.4, decreases slightly in the early epochs, and then remains relatively stable between 0.38 and 0.41, with minor fluctuations. This trend indicates that the model quickly learns the training data, while performance on the validation set stabilizes after the initial epochs. The observed gap between training and validation loss reflects differences in error on seen vs. unseen data, underscoring the importance of monitoring both metrics to assess model generalization.

**Figure 8:**
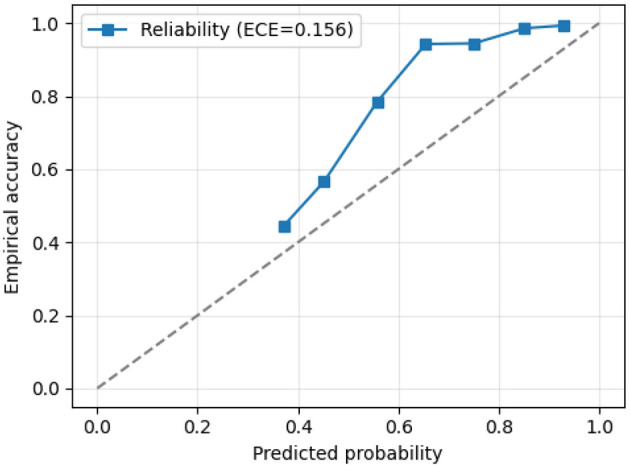
Reliability/calibration plot of the proposed EfficientOvaNet model.

Figure 8 presents the calibration plot, which conveys the alignment between predicted probabilities and actual outcomes. Proper calibration ensures the reliability of probabilistic predictions, which are crucial for clinical deployment and risk assessment. The *x*-axis represents the predicted probability (model confidence) ranging from 0 to 1, while the *y*-axis shows the empirical accuracy, i.e., the fraction of correct predictions at each confidence level. The dashed diagonal line indicates perfect calibration, where predicted probabilities match observed accuracies exactly (e.g., predictions with 0.7 confidence are correct 70% of the time). The plot line (blue with squares) illustrates the relationship between predicted probabilities and actual accuracies, showing slight underestimation at low confidence levels and modest overestimation at moderate to high confidence levels. The Expected Calibration Error (ECE) of 0.156 quantifies this deviation; lower values indicate better calibration. The reliability diagram is constructed using equal-width confidence bins. For each bin, empirical accuracy is computed as the fraction of correct predictions. The Expected Calibration Error (ECE) is calculated as the weighted average of the absolute difference between confidence and accuracy across all bins.

### 4.2 Phase 6: explainability, uncertainty, and clinician-in-the-loop analysis

The final phase focused on interpretability, uncertainty quantification, and clinician-in-the-loop validation to ensure transparency and clinical trustworthiness. Three complementary analyses, Grad-CAM visualization, Monte Carlo Dropout uncertainty estimation, and t-SNE feature embedding, were performed on the trained EfficientOvaNet model. Grad-CAM heatmaps, derived from the final convolutional block, localize salient regions that influence model predictions. Overlays confirmed that EfficientOvaNet consistently emphasized tumor regions rather than background noise. For uncertainty estimation, MC Dropout was re-enabled during inference to generate multiple stochastic forward passes, yielding mean confidence and variance-based uncertainty scores. Misclassified samples exhibited higher uncertainty, suggesting that uncertainty maps could guide radiologists toward cases requiring manual verification.

**Figure 9:**
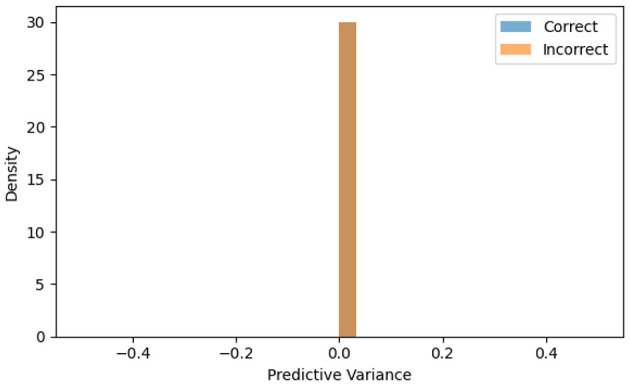
Uncertainty distribution of model predictions using Monte Carlo (MC) Dropout.

Figure 9 illustrates predictive uncertainty using MC Dropout. It shows the predictive uncertainty distribution using MC Dropout. Contrary to expectations, both correct and incorrect predictions exhibit near-zero variance, indicating that the MC Dropout implementation did not produce meaningful uncertainty estimates. This suggests limitations in using MC Dropout for uncertainty quantification in our architecture, as the model exhibits uniformly low variance across prediction accuracies. This represents a challenge for identifying ambiguous cases that would benefit from clinical review.

**Figure 10:**
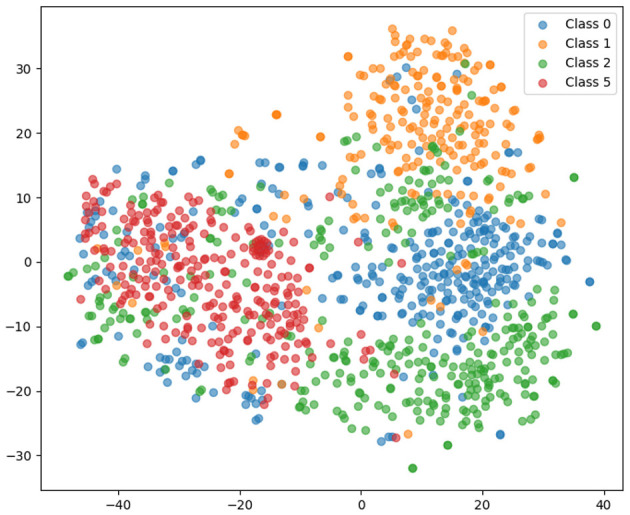
t-SNE visualization of high-dimensional feature embeddings extracted from the dual-branch EfficientOvaNet.

Figure 10 shows t-SNE visualization of high-dimensional feature embeddings. It demonstrates class separability in latent space, providing interpretability into how the model differentiates between tumor categories. Dense clusters indicate strong class discrimination, while overlaps identify challenging cases. In this, the data points are grouped into five tumor classes: class 0, class 1, class 2, class 3, and class 5, represented by blue, orange, green, purple, and red, respectively. The scatter plot displays these classes in a two-dimensional plane, revealing distinct clusters corresponding to different tumor categories. Dense groupings of the red and green classes in the left and bottom regions indicate strong class separability. In contrast, the orange and blue classes appear more dispersed toward the center and right, with slight overlaps suggesting challenging classification regions.

### 4.3 Comparison with baseline studies

To evaluate the effectiveness of the proposed EfficientOvaNet framework, we compared its performance with several baseline models reported in the literature on the MMOTU dataset. Table 3 summarizes the performance metrics, including accuracy, Dice coefficient, Intersection over Union (IoU), and F1-score. Previous studies primarily focused on segmentation and classification using U-Net variants, DeepLabV3+, PSPNet, DANet, and other deep learning architectures. For instance, Pham et al. (15) achieved a maximum accuracy of 71.65% using DANet on multi-class ovarian tumor segmentation, and PCU-Net (16) improved IoU and Dice by 4.23 and 2.99%, respectively, over the traditional U-Net. Deepika and Chitra (17) demonstrated that the U-Net with BCE-IoU loss achieved a Dice coefficient of 0.8234 and pixel-wise accuracy of 91.5%. The proposed EfficientOvaNet framework, which leverages a dual-branch EfficientNet-B3 architecture with ROI and global contextual features, outperforms these baseline models, achieving a mean accuracy of 91.9%, F1-score of 91.9%, and AUC of 0.98. This improvement can be attributed to the integration of global and local features, advanced preprocessing, data augmentation, class-imbalance control via weighted Focal Loss, and the use of explainable AI methods (Grad-CAM, Monte Carlo Dropout, and t-SNE) to enhance clinical interpretability. EfficientOvaNet demonstrates superior performance compared to prior work, indicating its potential to improve diagnostic accuracy and enable timely intervention in ovarian cancer management.

**Table 3:** Performance comparison of EfficientOvaNet with baseline studies on the MMOTU dataset.

**Method**	**Accuracy (%)**	**Dice**	**IoU**	**F1-score (%)**
DANet (15)	71.65	0.70	0.68	71.6
PCU-Net (16)	88.4	0.857	0.812	88.0
U-Net (BCE-IoU) (17)	91.5	0.8234	0.7592	91.5
EfficientOvaNet (proposed)	91.9	0.919	0.91	91.9

## 5 Conclusion

This research developed and validated EfficientOvaNet for classifying ovarian tumors from ultrasound images, using the MMOTU dataset. The model demonstrated high performance, with a mean accuracy of 91.9%, an F1-score of 91.9%, and an AUC of 0.98 in 5-fold cross-validation, outperforming traditional methods in terms of ultrasound variability and class imbalance. Transparency and clinical interpretability are achieved by combining Grad-CAM visualizations, Monte Carlo Dropout for uncertainty quantification, and t-SNE feature embeddings, thereby enhancing the trustworthiness of AI-assisted diagnostics. The results highlight the potential of EfficientOvaNet to transform ovarian cancer screening by enabling early, objective identification and reducing inter-observer variability in interpretation. This framework may allow timely interventions, optimize survival, and support resource-constrained environments, owing to its efficiency and cost-effectiveness in clinical settings. The shortcomings of the dataset, such as its emphasis on 2D ultrasound and the requirement for independent validation in other populations, are, however, limitations. Future research will focus on multimodal fusion (e.g., combining CEUS data), real-time implementation, and longitudinal studies to further enhance diagnostic reliability and address ethical issues related to AI in healthcare.

## Data Availability

The original contributions presented in the study are included in the article/supplementary material, further inquiries can be directed to the corresponding author.
